# Ca^2+^ overload- and ROS-associated mitochondrial dysfunction contributes to δ-tocotrienol-mediated paraptosis in melanoma cells

**DOI:** 10.1007/s10495-021-01668-y

**Published:** 2021-04-03

**Authors:** Michela Raimondi, Fabrizio Fontana, Monica Marzagalli, Matteo Audano, Giangiacomo Beretta, Patrizia Procacci, Patrizia Sartori, Nico Mitro, Patrizia Limonta

**Affiliations:** 1 Department of Pharmacological and Biomolecular Sciences, Università degli Studi di Milano, Milan, Italy; 2 Department of Environmental Science and Policy, Università degli Studi di Milano, Milan, Italy; 3 Department of Biomedical Sciences for Health, Università degli Studi di Milano, Milan, Italy

**Keywords:** Melanoma, Tocotrienols, Paraptosis, Mitochondrial impairment, ROS production, Ca^2+^ overload

## Abstract

**Abstract:**

Melanoma is an aggressive tumor with still poor therapy outcomes. δ-tocotrienol (δ-TT) is a vitamin E derivative displaying potent anti-cancer properties. Previously, we demonstrated that δ-TT triggers apoptosis in human melanoma cells. Here, we investigated whether it might also activate paraptosis, a non-canonical programmed cell death. In accordance with the main paraptotic features, δ-TT was shown to promote cytoplasmic vacuolization, associated with endoplasmic reticulum/mitochondrial dilation and protein synthesis, as well as MAPK activation in A375 and BLM cell lines. Moreover, treated cells exhibited a significant reduced expression of OXPHOS complex I and a marked decrease in oxygen consumption and mitochondrial membrane potential, culminating in decreased ATP synthesis and AMPK phosphorylation. This mitochondrial dysfunction resulted in ROS overproduction, found to be responsible for paraptosis induction. Additionally, δ-TT caused Ca^2+^ homeostasis disruption, with endoplasmic reticulum-derived ions accumulating in mitochondria and activating the paraptotic signaling. Interestingly, by using both IP3R and VDAC inhibitors, a close cause-effect relationship between mitochondrial Ca^2+^ overload and ROS generation was evidenced. Collectively, these results provide novel insights into δ-TT anti-melanoma activity, highlighting its ability to induce mitochondrial dysfunction-mediated paraptosis.

**Graphic Abstract:**

δ-tocotrienol induces paraptotic cell death in human melanoma cells, causing endoplasmic reticulum dilation and mitochondrial swelling. These alterations induce an impairment of mitochondrial function, ROS production and calcium overload.
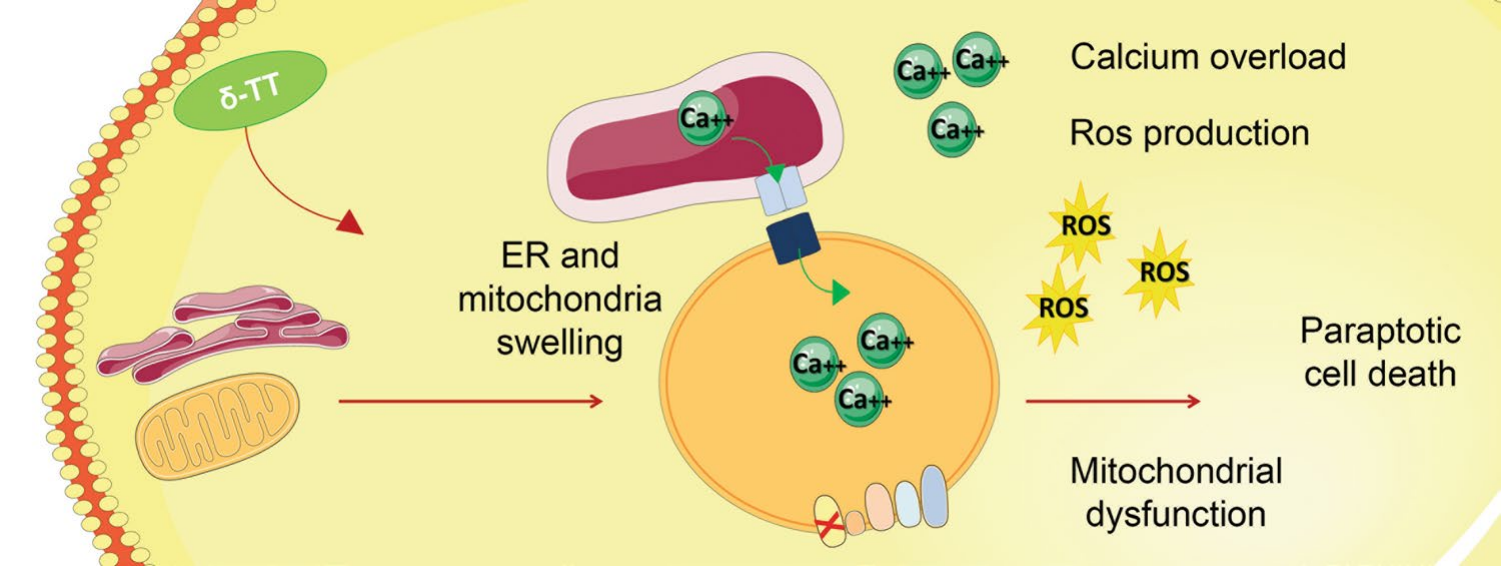

## Introduction

Cutaneous melanoma is the third most common type and the deadliest form of skin cancer [1]. Surgical resection represents the first-choice treatment when melanoma is diagnosed at an early phase; 5-year survival rates range from 82 to 98% for stages I-II [2]. On the other hand, advanced melanomas (stages III and IV) are highly aggressive, and currently available therapeutic strategies include chemotherapy (i.e. dacarbazine), targeted therapy (i.e. vemurafenib, dabrafenib, trametinib, encorafenib, binimetinib and cobimetinib) and immunotherapy (i.e. ipilimumab, pembrolizumab, nivolumab and atezolizumab). Unfortunately, these approaches are characterized by a low success rate due to the development of drug resistance [3–7]. In addition, most of these treatments are often accompanied by severe side effects [8, 9]. In this context, more effective and better-tolerated anti-melanoma options need to be urgently identified.

Paraptosis was first observed by Sperandio and collaborators in 2000 [10]. It consists of a programmed cell death displaying cytoplasmic vacuolization, generally associated with endoplasmic reticulum (ER) and/or mitochondrial swelling. Since paraptosis requires protein synthesis, it can be inhibited by cycloheximide, an inhibitor of translation [11]. In contrast to apoptosis, paraptosis does not involve caspase cleavage or apoptotic body formation, but it has been found to be frequently mediated by mitogen-activated protein kinase (MAPK) family members, such as c-Jun N-terminal protein kinase 1 (JNK1), P38 and mitogen-activated protein kinase kinase 2 (MEK-2) [12]. Moreover, it is often accompanied by protein misfolding and ER stress, as well as by an alteration of Ca^2+^ and redox homeostasis [11, 13, 14]. In particular, a key role is played by mitochondria-associated ER membranes (MAMs), which facilitate the Ca^2+^ flux from the ER to mitochondria, leading to the oxidative metabolism impairment and the following ROS overproduction frequently observed in case of pro-paraptotic Ca^2+^ overload [11, 13, 14]. Intriguingly, paraptosis-inducing compounds have been shown to overcome cancer multi-drug resistance to apoptosis [15–17].

Tocotrienols (TTs) are vitamin E derivatives displaying potent anti-tumor properties [18–22]. We previously demonstrated that the δ isomer could induce ER stress-related apoptosis in A375 and BLM human melanoma cells, while sparing normal melanocytes [23]. Moreover, it has been recently reported to trigger paraptosis in colon and prostate cancer cells [24–26]. However, the ability of δ-TT to specifically activate paraptotic cell death in melanoma has not been investigated yet.

In this study, we further dissected the molecular mechanisms underlying the growth-suppressing activity of δ-TT in human melanoma cells, with special regard to its ability to promote paraptosis-associated biochemical changes, including impairment of mitochondrial function and perturbation of intracellular Ca^2+^ and redox homeostasis.

## Materials and methods

### Cell cultures

A375 human melanoma cell line was purchased from American Type Culture Collection (Manassas, VA, USA). BLM human melanoma cell line was kindly provided by Dr. G.N. van Muijen from Radbound University Nijmegen Medical Center (Department of Pathology, Nijmegen, The Netherlands). Cells were stored in liquid nitrogen and, after resuscitation, they were kept in culture for 10–12 weeks. In particular, they were cultured in humidified atmosphere of 5% CO2/95% air at 37  °C, in DMEM medium supplemented with antibiotics, glutamine and 7.5% (A375 cells) or 10% (BLM cells) FBS. Cell line authenticity was assessed by the Short Tandem Repeat (STR) profile analysis, as explained by ATCC Standards Development Organization (SDO) in ANSI Standard (ASN-0002).

### Chemicals and antibodies

δ-TT was extracted from Annatto seeds (*Bixa orellana*) from American River Nutrition Inc. (Hadley, MA, USA) [27], and it was used at the concentration of 15 μg/mL in all the experiments. The following antibodies from Cell Signaling Technology Inc. (Danvers, MA, USA) were used for western blot analysis: pAMPK (2535), AMPK (5832), pJNK (4668), JNK (9252), pP38 (4511), p38 (8690), pERK1/2 (4370) ERK1/2 (4695). α-Tubulin (T6199) was from Sigma-Aldrich (Milano, Italy), and Total OXPHOS (ab110411) was from Abcam (Cambridge, UK). Horseradish-peroxidase-conjugated secondary antibodies were from Cell Signaling Technology, and enhanced chemiluminescence solution was from Cyanagen (Bologna, Italy). The pan‐caspase inhibitor Z‐VAD‐FMK was from R&D System Inc (Minneapolis, MN, USA). Cycloheximide, the inhibitor of protein synthesis, 2APB (2-Aminoethyl diphenylborinate), the inhibitor of IP3R, DIDS (disodium 4,4′-diisothiocyanostilbene-2,2′-disulfonate), the inhibitor of VDAC, and NAC (N-acetyl-l- cysteine), the ROS scavenger, were purchased from Sigma-Aldrich. MitoTracker Orange CMTMRos, MitoSOX Red, Fluo-3 AM and Rhod-2 AM were purchased from Thermo Fisher Scientific Inc. (Monza, Italy).

### Morphological analysis

Cells were treated with δ-TT or pretreated with NAC, 2APB or DIDS and then with δ-TT. After 24 h, cell morphology was analyzed by light microscopy. A Zeiss Axiovert 200 microscope was used for the analysis, and cells were observed with a 20 × 1.4 objective lens connected to a Coolsnap Es CCD camera (Roper Scientific-Crisel Instruments, Roma, Italy).

### Electron microscopy

Cell were fixed o/n in a solution of 0.1 M sodium cacodylate buffer (pH 7.4) with 2% paraformaldehyde and 2% glutaraldehyde. They were washed in the same buffer for 45 min and post-fixed in 0,2 M cacodylate buffer with 1% osmium tetroxide at 0 °C for 90 min. Pellets were then rinsed in distilled water, stained en block with 2% aqueous uranyl acetate, dehydrated in acetone and embedded in Epon-Araldite resin. A Leica Supernova ultramicrotome (Reichert Ultracut E) was used to cut ultrathin sections. Sections were collected on 100-mesh grids and counterstained with lead citrate. Transmission electron microscopy was performed with a Zeiss EM10 electron microscope (Carl Zeiss, Oberkochen, Germany).

### Western blotting

After treatment, melanoma cells were lysed in RIPA buffer, and samples were resolved on SDS-PAGE. Proteins were then transferred to a nitrocellulose membrane. Membranes were incubated with the specific primary and secondary antibodies. Westar EtaC Ultra 2.0 chemiluminescence solution (Cyanagen, Bologna, Italy) was used for the detection. α-Tubulin was used as a housekeeping protein control.

### Oxygen consumption

To measure oxygen consumption rate (OCR), a Clark oxygen electrode—DW1 electrode chamber—was used (Hansatech Instruments Ltd, Norfolk, UK). Treated cells were rinsed in PBS and then suspended in coupled respiration buffer containing Na-pyruvate (1 mM), d-glucose (25 mM), 40 μg/mL digitonin, 2% free-fatty acid BSA or electron flow buffer containing Na-pyruvate (10 mM), 40 μg/mL digitonin, 2% free-fatty acid BSA, malate (2 mM), carbonyl cyanide m-chlorophenyl hydrazine (CCCP, 4 μM). Cells were then transferred to the electrode chamber, and basal, uncoupled (adding oligomycin 10 µM) and maximal respiration (adding CCCP 10 µM) was measured.

### Flow cytometry analysis

After treatment, cells were incubated with MitoTracker Orange CMTMRos (10 nM, 30 min) to assess mitochondrial activity; with MitoSOX Red (10 µM, 10 min) to quantify mitochondrial ROS production; with Fluo-3 AM and Rhod-2 AM (5 µM, 30 min) to measure cytosolic and mitochondrial Ca^2+^ levels. To perform flow cytometry analyses, Novocyte3000 instrument from ACEA Biosciences (San Diego, CA, USA) was used. Data were analyzed with Novoexpress software.

### ATP measurement

The effect of δ-TT treatment on ATP production in melanoma cells was assessed by using an ATP colorimetric assay kit purchased from GeneTex (Alton Pkwy Irvine, CA, USA), and the detection was done through an EnSpire Multimode Plate reader purchased from PerkinElmer (Milano, Italy).

### MTT assay

A375 and BLM human melanoma cells were seeded in 24-well plates, pretreated with NAC, 2APB or DIDS and then with δ-TT. After treatment, medium was replaced with DMEM w/o phenol red and w/o FBS, containing 0.5 mg/mL of 3-(4,5-dimethylthiazole-2-yl)-2,5-diphenyltetrazolium bromide (MTT). Isopropanol was used to dissolve the violet crystals, and absorbance at 550 nm was measured through an EnSpire Multimode Plate reader.

### Statistical analysis

A GraphPad Prism5 from GraphPad Software (San Diego, CA) was utilized for statistical analysis. Data represent the mean ± SEM of three-four different experiments. To determine the differences between sample groups, *T*-test or one-way analysis of variance (ANOVA) were performed, followed by Bonferroni’s test in the case of ANOVA. The differences between the groups were considered statistically significant when P value was < 0.05.

## Results

### δ-TT induces paraptosis in human melanoma cells

Paraptosis is a non-canonical cell death known to involve cytoplasmic vacuolation, usually associated with ER/mitochondrial swelling, and to be dependent on protein synthesis as well as on MAPK activation [10, 12].

**Fig. 1:**
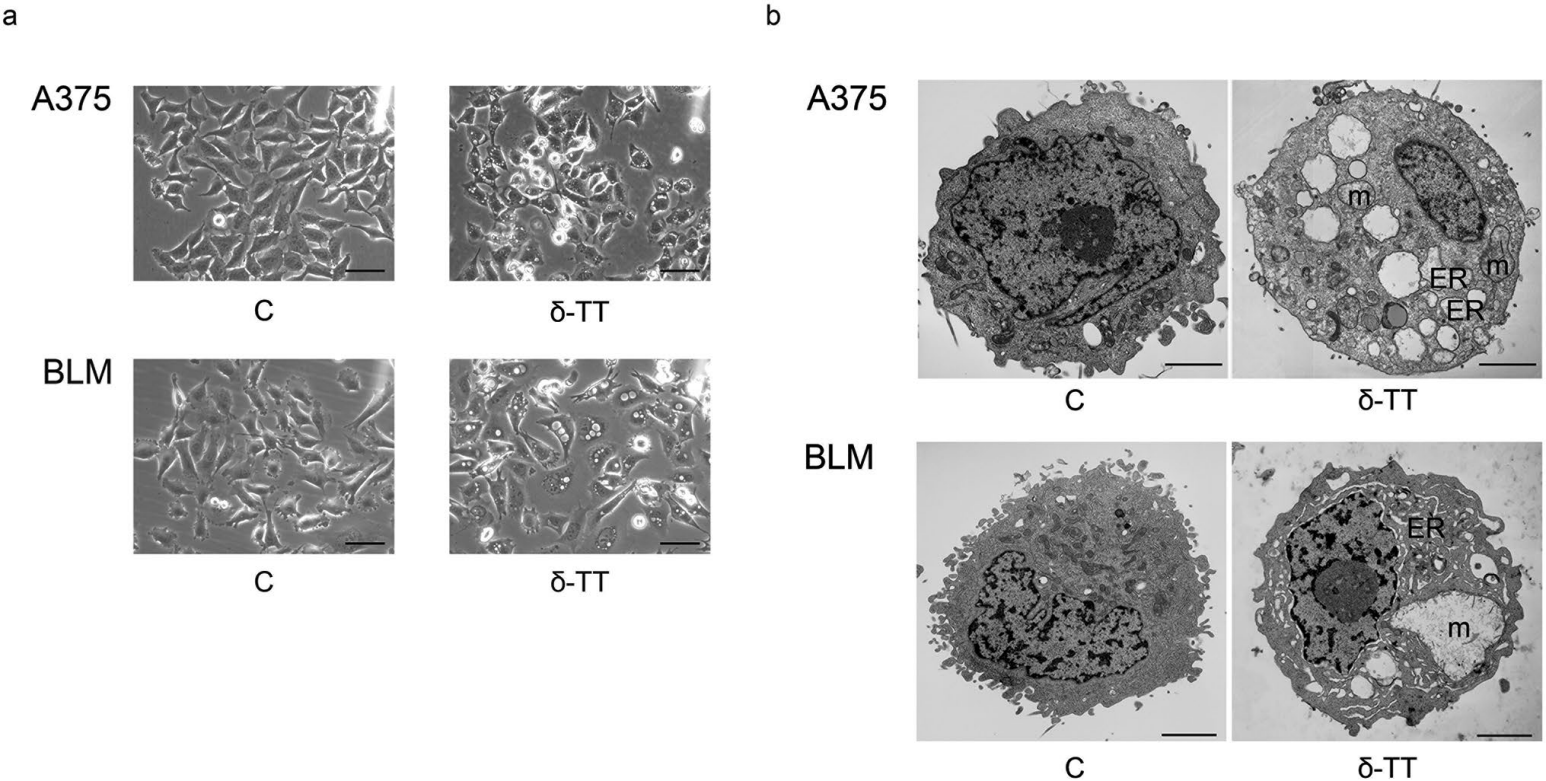
δ-TT induces cytoplasmic vacuolization in human melanoma cells. **a** A375 and BLM cells were treated with 15 μg/mL δ-TT for 12 h and then analyzed by light microscopy, showing extensive cytoplasmic vacuolization. Scale bars are 20 µm. **b** A375 and BLM cells were treated with 15 μg/mL δ-TT for 12 h and then analyzed by electron microscopy. They exhibited swollen mitochondria with rare cristae (m), dilated endoplasmic reticulum (ER) cisternae and enlarged nuclear envelope. Scale bars are 2 µm

**Fig. 2:**
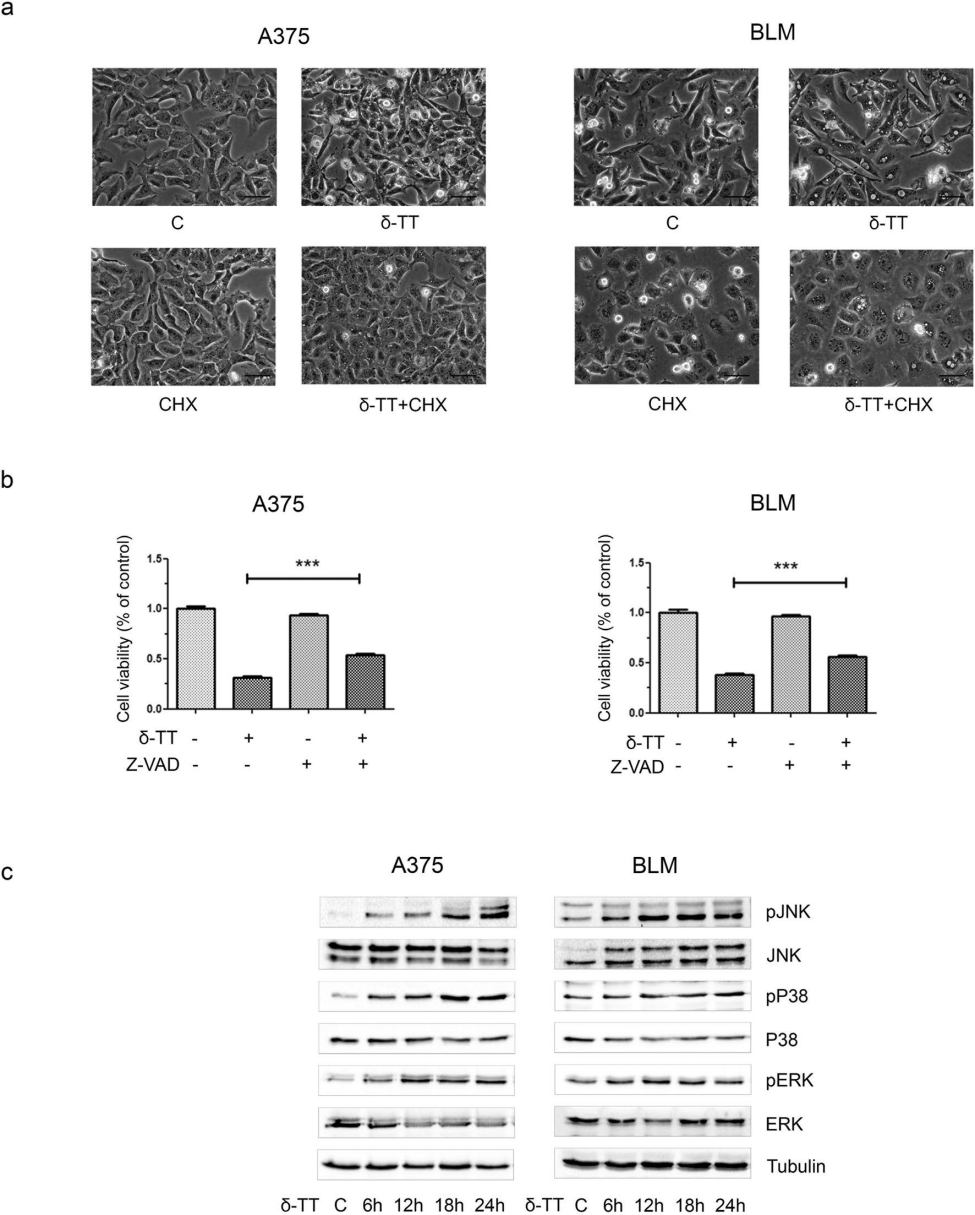
δ-TT induces paraptotic cell death in human melanoma cells. **a** A375 and BLM cells were incubated with the translation inhibitor cycloheximide (CHX) (20 µM) 4 h before treatment with δ-TT (15 µg/mL, 12 h) and observed under light microscopy. Images show that CHX suppressed δ-TT-induced vacuolization in melanoma cells. Scale bars are 20 µm. **b** A375 and BLM cells were incubated with 50 μM Z-VAD-FMK, the pan-caspase inhibitor, for 4 h and then treated with 15 μg/mL δ-TT for 24 h. MTT assay was performed to assess melanoma cell viability. Three experiments have been performed. One-way analysis of variance. Post-test: Bonferroni’s test. Mean values  ±  SEM are showed. ***P < 0.001 versus controls. **c** A375 and BLM cells were treated with 15 µg/mL δ-TT for 6–24 h. To evaluate the expression levels of pJNK, pP38 and pERK1/2, western blot analysis was performed. Tubulin was used as a housekeeping protein control. Three experiments have been performed

Herein, we demonstrated that δ-TT (15 μg/mL, 12 h) can trigger extensive vacuolation in A375 and BLM cell lines (Fig. 1a). In particular, as evidenced by TEM analysis, δ-TT-treated cells exhibited dilation of ER cisternae and swollen mitochondria, with both organelles displaying a severe alteration of their architecture. Moreover, in BLM cells the nuclear envelope appeared expanded (Fig. 1b). Since paraptosis is known to be dependent on protein synthesis, it can be inhibited by cycloheximide, a translation inhibitor; on the other hand, it is not modulated by caspases, thus it cannot be blocked by inactivation of these proteins. Notably, pretreatment of melanoma cells with cycloheximide (20 μM, 4 h) significantly prevented δ-TT mediated vacuole formation (Fig. 2a), supporting the strict link between the latter and protein synthesis; on the contrary, pretreatment of A375 and BLM cells with the pan-caspase inhibitor Z-VAD-FMK only partially counteracted δ-TT related cytotoxicity, confirming the induction of an alternative cell death mechanism besides apoptosis (Fig. 2b). Finally, δ‐TT (15 μg/mL, 24 h) was found to induce a time-dependent activation (phosphorylation) of JNK, P38 and ERK1/2 in both melanoma cell lines (Fig. 2c). Collectively, these data indicate that δ‐TT exerts pro-paraptotic effects on both A375 and BLM cells, inducing an extensive alteration of ER and mitochondrial structure and activating the MAPK molecular pathway.

### δ-TT impairs mitochondrial metabolism in melanoma cells

**Fig. 3:**
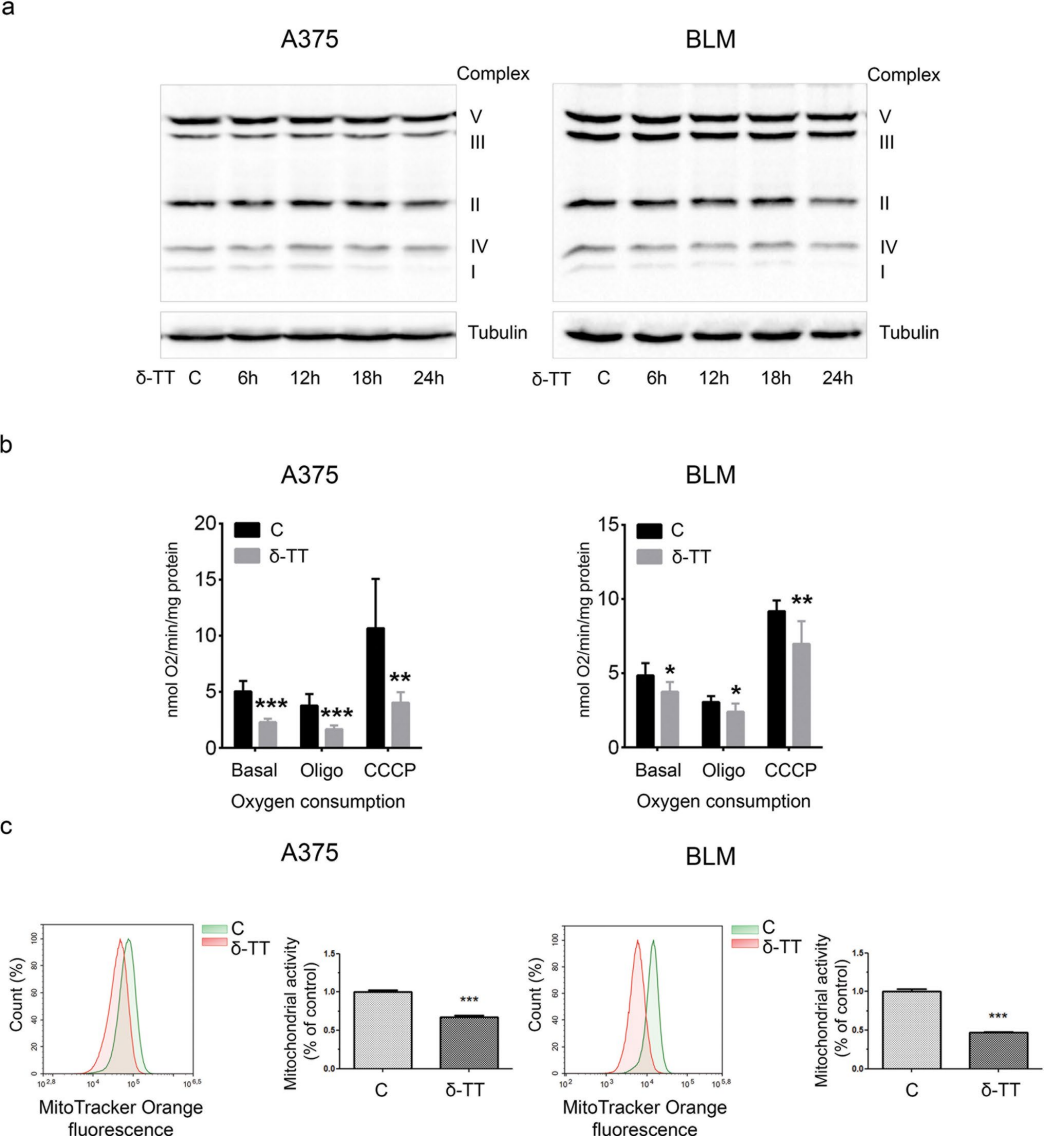
δ-TT treatment affects mitochondrial function in melanoma cells. **a** A375 and BLM cells were treated with 15 µg/mL δ-TT for 6–24 h. OXPHOS expression levels were evaluated via western blot analysis. Tubulin was used as a housekeeping protein control. Three experiments have been performed. **b** A375 and BLM cells were treated with 15 µg/mL δ-TT for 12 h. Clark electrode was used to measure oxygen consumption. Analysis was performed in basal respiration conditions, in uncoupled respiration conditions (adding oligomycin, oligo) and in maximal respiration conditions (adding carbonyl cyanide m-chlorophenyl hydrazine, CCCP). Three experiments have been performed. T-test. Mean values  ±  SEM are shown. *P < 0.05 versus controls; **P < 0.01 versus controls; ***P < 0.001 versus controls. **c** A375 and BLM cells were treated with 15 µg/mL δ-TT for 12 h. Cells were stained with MitoTracker Orange CMTM Ros (10 nM, 30 min) fluorescent probe. Mitochondrial activity was measured by flow cytometry. Three experiments have been performed. T-test. Mean values  ±  SEM are shown. ***P < 0.001 versus controls

**Fig. 4:**
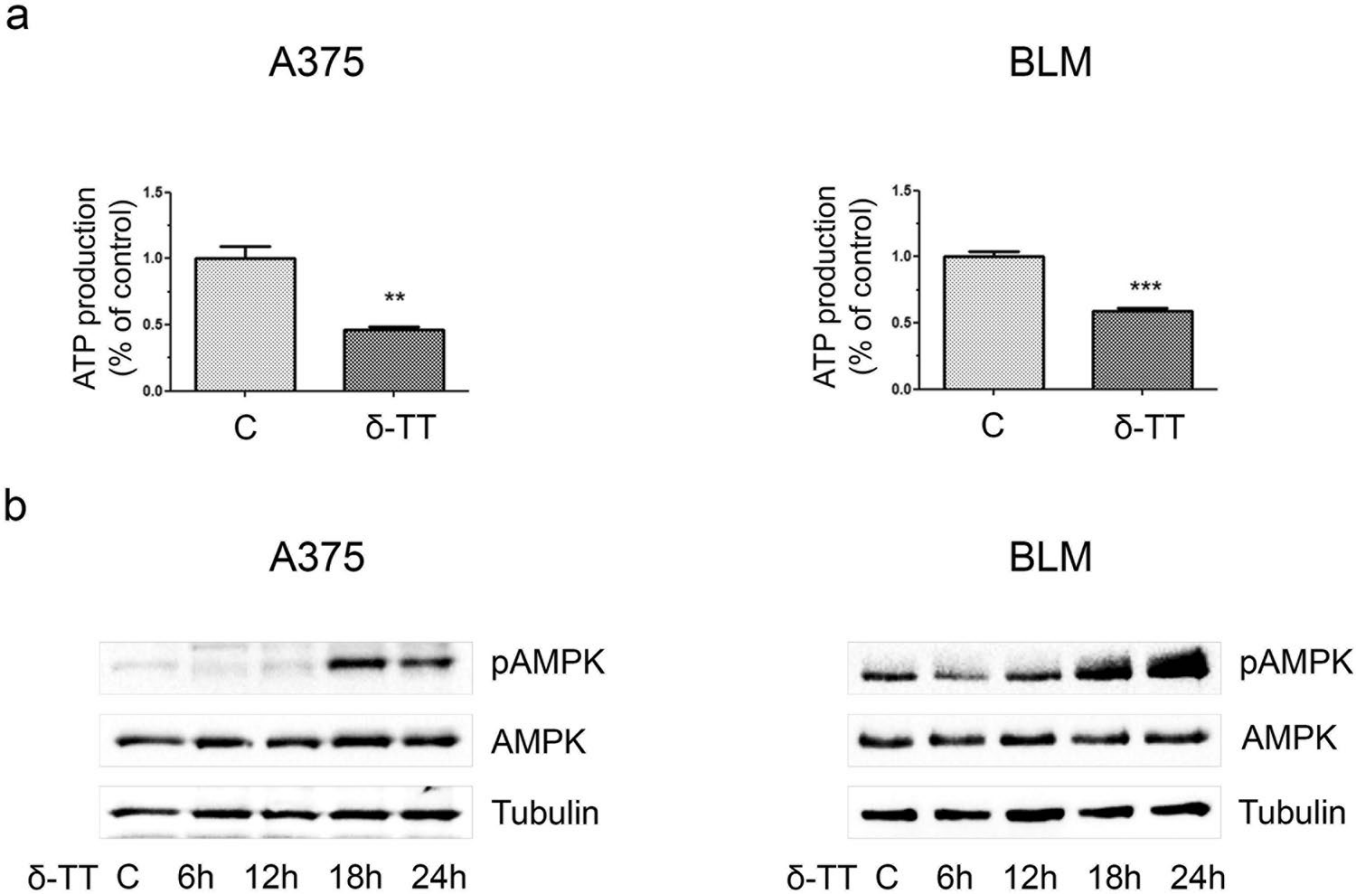
δ-TT causes ATP depletion in human melanoma cells. **a** A375 and BLM cells were treated with 15 µg/mL δ-TT for 12 h. ATP colorimetric assay was used to measure ATP production levels. Three experiments have been performed. T-test. Mean values  ±  SEM are shown. **P < 0.01 versus controls; ***P < 0.001 versus controls. **b** A375 and BLM cells were treated with 15 µg/mL δ-TT for 6−24 h. Expression levels of pAMPK were assessed via western blot analysis. Tubulin was used as a housekeeping protein control. Three experiments have been performed

Given the morphological damage caused by δ-TT to the mitochondria of A375 and BLM cell lines, we further investigated if the compound could alter the function of these organelles and thus impair the cellular oxidative metabolism. Figure 3a shows that δ-TT treatment (15 μg/mL, 24 h) of melanoma cells resulted in a time-dependent downregulation of oxidative phosphorylation (OXPHOS) protein complex I, accompanied by a reduction of O_2_ consumption (12 h) in basal, uncoupled (adding 10 μM oligomycin) and maximal (adding 10 μM carbonyl cyanide m-chlorophenyl hydrazine, CCCP) respiration conditions (Fig. 3b). This alteration of the oxidative metabolism caused by δ-TT resulted in a rapid loss of mitochondrial membrane potential (MMP) (12 h), reflecting a reduction in mitochondrial activity, as shown by flow cytometric analysis after incubation with 10 nM MitoTracker Orange CMTMRos for 30 min (Fig. 3c). As a direct consequence of this mitochondrial dysfunction, a significant decrease in ATP production (12 h) and a parallel phosphorylation of the energy sensor AMPK (18–24 h) were observed (Fig. 4a,b), highlighting the ability of δ-TT to exert energy-depleting effects on melanoma cells. Taken together, these data point out that δ-TT markedly alters OXPHOS and energy homeostasis in both A375 and BLM cell lines.

### Mitochondrial ROS overproduction is implicated in the pro-paraptotic effects of δ-TT on human melanoma cells

It is now well established that high mitochondrial ROS levels can be responsible for the induction of paraptotic cell death, with several natural compounds triggering this pathway in cancer cells via mitochondrial dysfunction-associated oxidative stress [11].

**Fig. 5:**
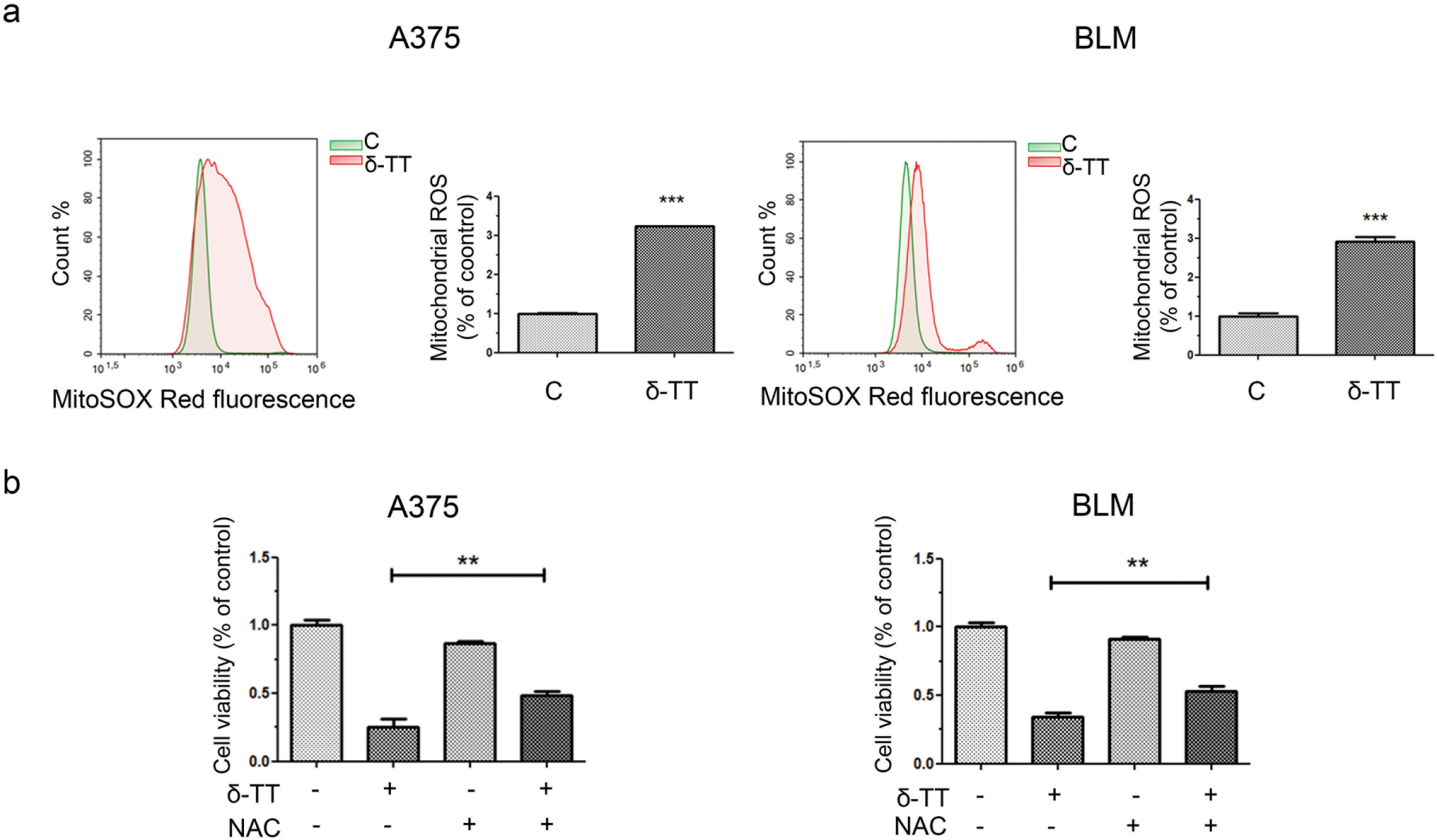
δ-TT induces mitochondrial ROS overproduction in human melanoma cells. **a** A375 and BLM cells were treated with 15 µg/mL δ-TT for 12 h and then stained with MitoSOX Red (5 µM, 10 min) fluorescent probe. ROS generation in mitochondria was assessed by flow cytometry. T-test. Mean values ± SEM are shown. ***P < 0.001 versus controls. **b** A375 and BLM cells were incubated with 5 mM NAC (N-acetyl-l-cysteine), the ROS scavenger, for 2 h and then treated with 15 μg/mL δ-TT for 24 h. MTT assay was performed to assess melanoma cell viability. Three experiments have been performed. One-way analysis of variance. Post-test: Bonferroni’s test. Mean values  ±  SEM are shown. **P < 0.01 versus controls

**Fig. 6:**
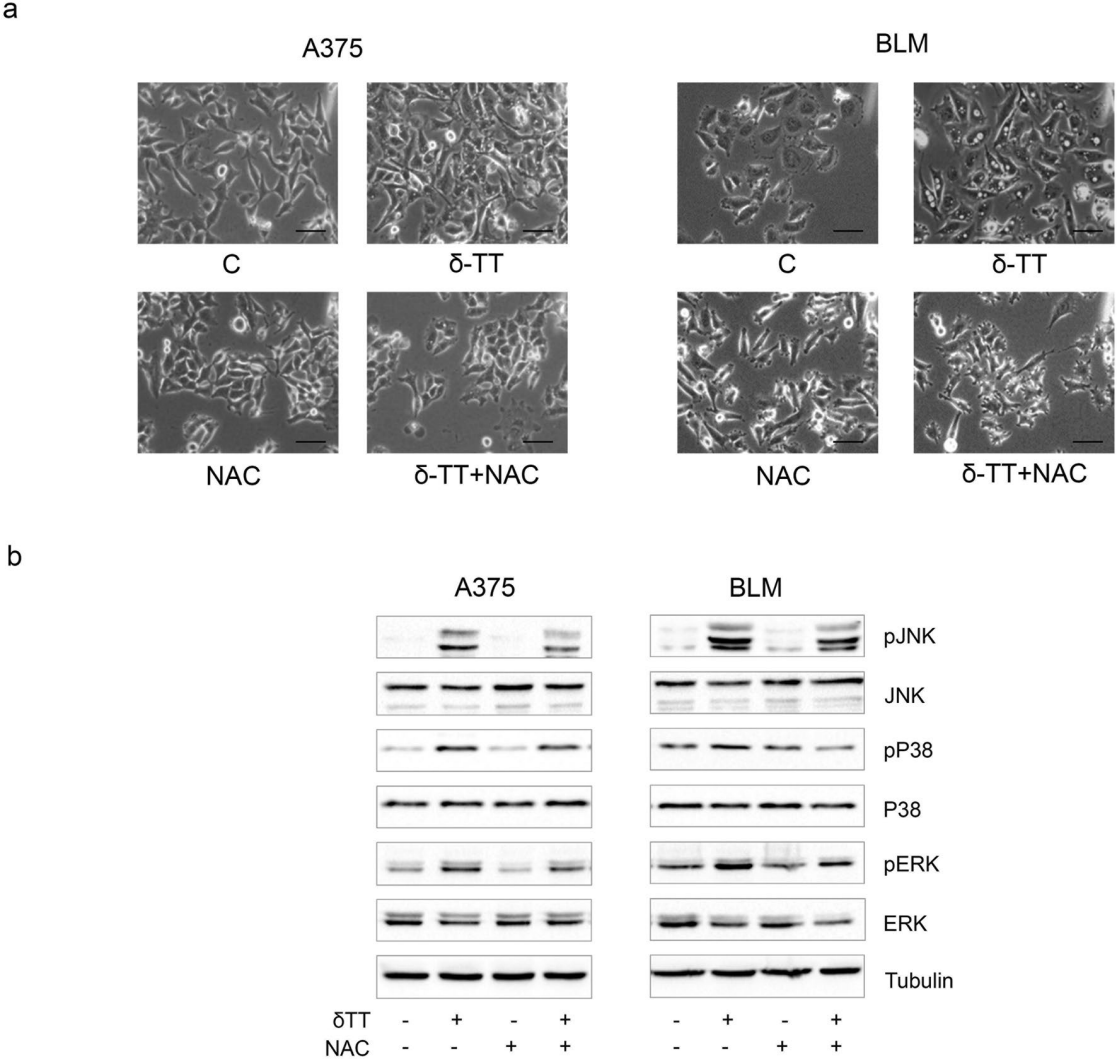
δ-TT-induced ROS are involved in paraptotic vacuolization and MAPK activation. **a** A375 and BLM cells were incubated with 5 mM NAC for 2 h and then treated with 15 μg/mL δ-TT for 12 h. Cell morphology was analyzed by light microscopy. Scale bars are 20 µm. **b** A375 and BLM cells were incubated with 5 mM NAC for 2 h and then treated with 15 μg/mL δ-TT for 24 h. The expression levels of pJNK, pP38 and pERK1/2 were evaluated via western blot analysis. Tubulin was used as a housekeeping protein control. Three experiments have been performed

Herein, we measured ROS production in melanoma cells treated with δ-TT (15 μg/mL). A three-fold increase in mitochondrial ROS production was found (12 h), as evidenced by flow cytometric analysis after staining with MitoSOX Red (Fig. 5a). By using the antioxidant NAC (5 mM, 2 h), we then assessed the role of ROS in δ-TT-related paraptosis: interestingly, NAC pretreatment markedly rescued melanoma cell viability (24 h) (Fig. 5b), also suppressing cytoplasmic vacuolation (12 h) and MAPK phosphorylation (24 h) (Fig. 6a,b). Hence, we demonstrated that mitochondrial oxidative stress is deeply implicated in the pro-paraptotic effects of δ-TT in melanoma cells.

### Mitochondrial Ca^2+^ overload causes δ-TT-associated paraptosis in melanoma cells

**Fig. 7:**
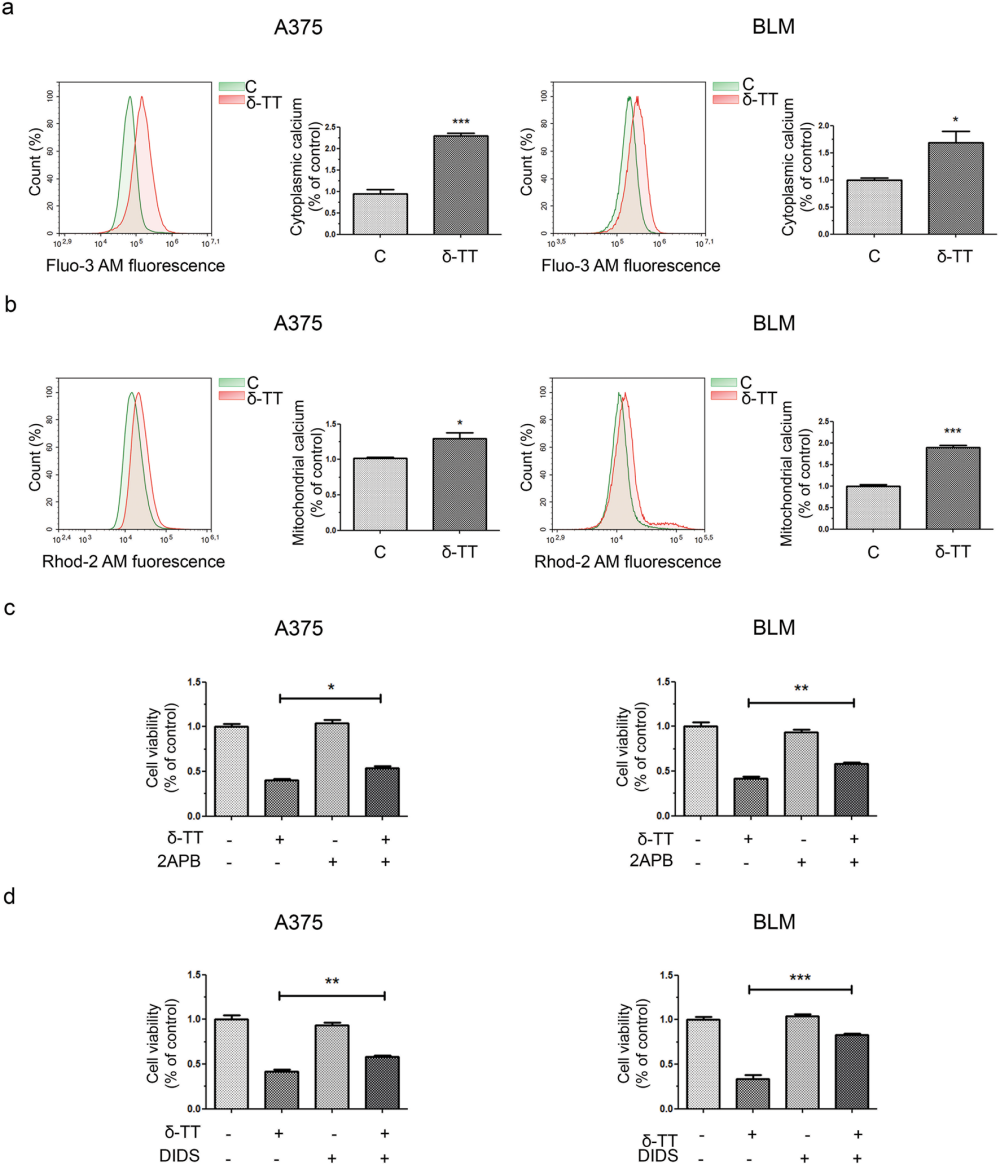
δ-TT causes Ca^2+^ homeostasis dysregulation in human melanoma cells. **a** A375 and BLM cells were treated with 15 μg/mL δ-TT for 12 h and then stained with 5 µM Fluo-3 AM for 30 min. Cytoplasmic Ca^2+^ levels were assessed by flow cytometry. Three experiments have been performed. T-test. Mean values  ±  SEM are shown. *P < 0.05 versus controls; ***P < 0.001 versus controls. **b** A375 and BLM cells were treated with 15 μg/mL δ-TT for 12 h and then stained with 5 µM Rhod-2 AM for 30 min. Mitochondrial Ca^2+^ levels were evaluated by flow cytometry. Three experiments have been performed. T-test. Mean values  ±  SEM are shown. *P < 0.05 versus controls; ***P < 0.001 versus controls. **c** A375 and BLM cells were incubated with the IP3R inhibitor 2APB (20 µM, 2 h) and then treated with 15 μg/mL δ-TT for 24 h. MTT assay was performed to evaluate cell viability. Three experiments have been performed. Mean values  ±  SEM are shown. One-way analysis of variance. Post-test: Bonferroni’s test. *P < 0.05 versus controls; **P < 0.01 versus controls. **d** A375 and BLM cells were incubated with the VDAC blocker DIDS (75 µM, 2 h) and then treated with 15 μg/mL δ-TT for 24 h. MTT assay was performed to evaluate cell viability. Three experiments have been performed. Mean values  ±  SEM are shown. One-way analysis of variance. Post-test: Bonferroni’s test. **P < 0.01 versus controls; ***P < 0.001 versus controls

**Fig. 8:**
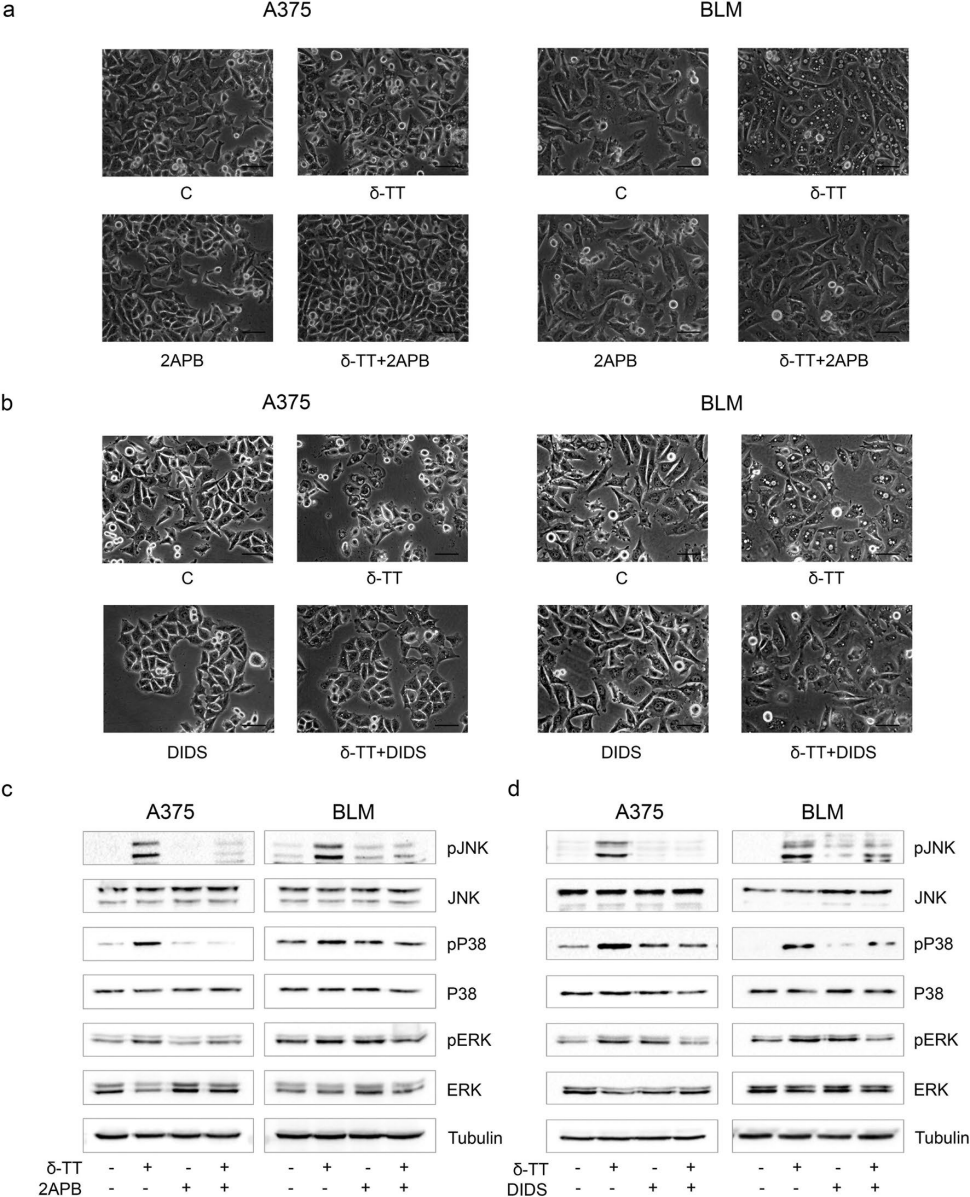
δ-TT-induced Ca^2+^ overload is involved in paraptotic vacuolization and MAPK activation. **a** A375 and BLM cells were incubated with the IP3R inhibitor 2APB (20 µM, 2 h) before treatment with 15 μg/mL δ-TT for 12 h. Cell morphology was analyzed via light microscopy. Scale bars are 20 µm. **b** A375 and BLM cells were incubated with the VDAC blocker DIDS (75 µM, 2 h) before treatment with 15 μg/mL δ-TT for 12 h. Cell morphology was analyzed via light microscopy. Scale bars are 20 µm. **c** A375 and BLM cells were incubated with the IP3R inhibitor 2APB (20 µM, 2 h) before treatment with 15 μg/mL δ-TT for 24 h. The expression levels of pJNK, pP38 and pERK1/2 were assessed via western blot. Tubulin was used as a housekeeping protein control. Three experiments have been performed. **d** A375 and BLM cells were incubated with DIDS (75 µM, 2 h) before treatment with 15 μg/mL δ-TT for 24 h. The expression levels of pJNK, pP38 and pERK1/2 were assessed via western blot analysis. Tubulin was used as a housekeeping protein control. Three experiments have been performed

**Fig. 9:**
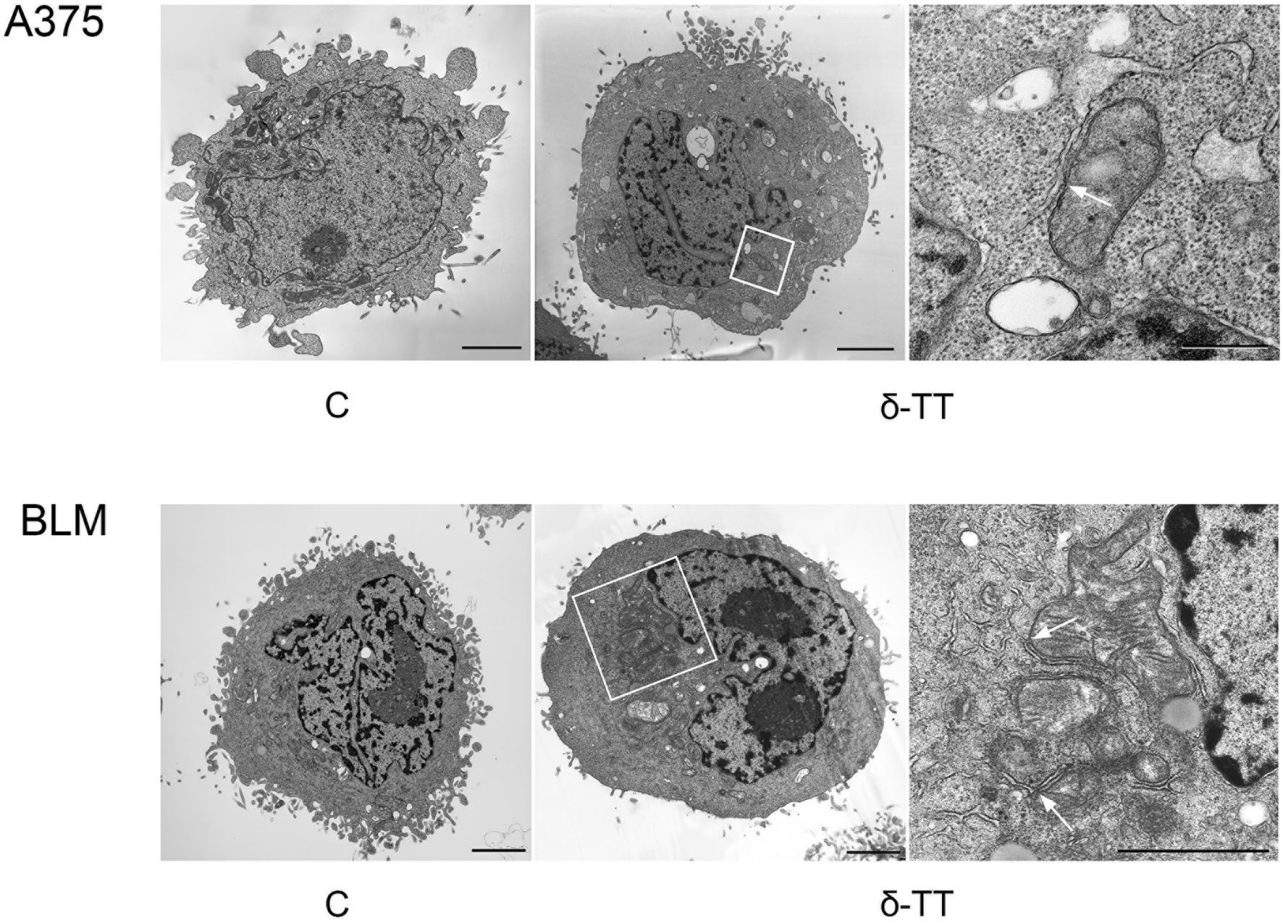
Formation of MAMs is observed after δ-TT treatment. A375 and BLM cells were treated with 15 μg/mL δ-TT for 12 h and subsequently analyzed via electron microscopy. Boxed areas in central panels show the formation of MAMs after δ-TT treatment. Scale bars are 2 μm. Boxed areas are enlarged in the right panels and MAMs are highlighted by arrows. Scale bars are 0,5 μm (upper panel) and 2 μm (lower panel)

ER and mitochondria are the main reservoirs of Ca^2+^ inside the cell, and a damage to these structures can cause a dysregulation of ion homeostasis, resulting in intracellular and mitochondrial Ca^2+^ overload. Different anti-cancer compounds triggering ER stress-mediated cell death have been reported to induce the release of Ca^2+^ from ER and its subsequent uptake into mitochondria [28–32]. In particular, Ca^2+^ is released from ER via inositol trisphosphate receptor (IP3R) and enters mitochondria through the voltage-dependent anion channel (VDAC), located on the outer mitochondrial membrane [33]. Paraptotic cell death induced by several nutraceuticals in tumors has been reported to frequently correlate with Ca^2+^ overload [11]; therefore, we investigated the effect of 15 μg/mL δ-TT on the ion homeostasis of A375 and BLM cells. We showed that the compound can significantly increase both cytoplasmic and mitochondrial (5 µM Fluo-3 AM 30 min and 5 µM Rhod-2 AM 30 min, respectively) Ca^2+^ levels in melanoma cell lines (12 h) (Fig. 7a, b). To evaluate the role of Ca^2+^ overload in δ-TT-associated paraptosis, we pretreated melanoma cells with 2APB (20 µM, 2 h), an inhibitor of IP3R, as well as with DIDS (75 µM, 2 h), a blocker of VDAC. Both these molecules successfully counteracted δ-TT (15 μg/mL, 24 h) cytotoxic activity (Fig. 7c, d), preventing vacuole formation (12 h) (Fig. 8a, b) and JNK, P38 and ERK1/2 phosphorylation (24 h) (Fig. 8c, d). These results suggest that ER-stored Ca^2+^ contributes to the mitochondrial swelling and subsequent paraptotic cascade observed in δ-TT-treated A375 and BLM cells. Indeed, further TEM analysis revealed the presence of MAMs, specific ER-mitochondria contact sites, in both these cell lines (Fig. 9), supporting the existence of Ca^2+^ fluxes at the ER-mitochondria interface.

### δ-TT triggers paraptosis in melanoma cells via the Ca^2+^/ROS axis

**Fig. 10:**
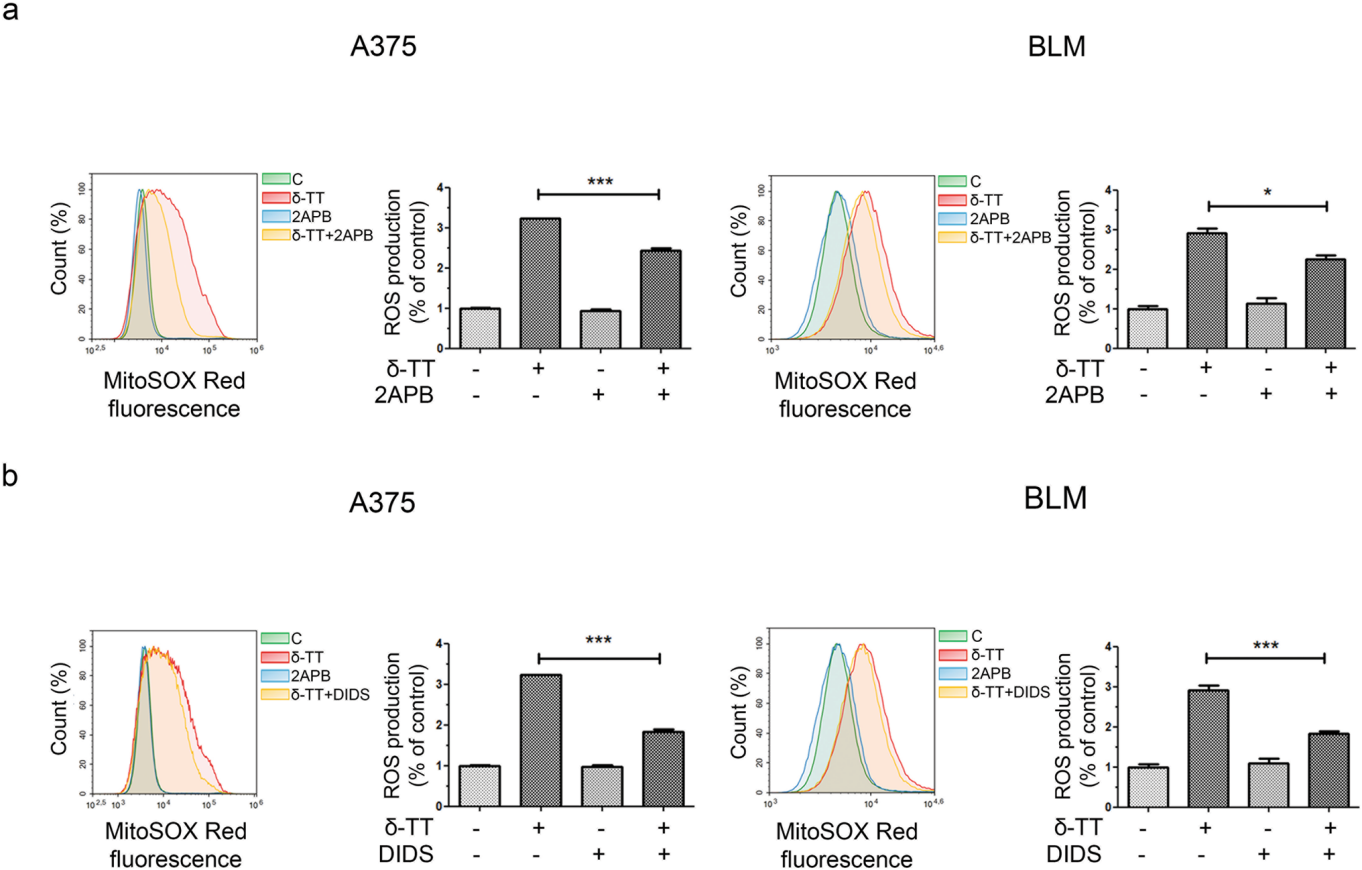
δ-TT-induced Ca^2+^ overload causes mitochondrial ROS overproduction in human melanoma cells. **a** A375 and BLM cells were incubated with the IP3R inhibitor 2APB (20 µM, 2 h) before treatment with 15 μg/mL δ-TT for 12 h. Then, cells were stained with MitoSOX Red (5 µM, 10 min) fluorescent probe. Mitochondrial ROS generation was measured by flow cytometry. Three experiments have been performed. Mean values  ±  SEM are shown. One-way analysis of variance. Post-test: Bonferroni’s test. *P < 0.05 versus controls; ***P < 0.001 versus controls. **b** A375 and BLM cells were incubated with the VDAC blocker DIDS (75 µM, 2 h) before treatment with 15 μg/mL δ-TT for 12 h. Then, cells were stained with MitoSOX Red (5 µM, 10 min) fluorescent probe. Mitochondrial ROS generation was measured by flow cytometry. Three experiments have been performed. Mean values ± SEM are shown. One-way analysis of variance. Post-test: Bonferroni’s test. ***P < 0.001 versus controls

Nutraceutical-stimulated mitochondrial Ca^2+^ overload has been directly linked to ROS-mediated paraptosis in different tumor types [11]. Thus, we investigated the interconnection between ROS levels and Ca^2+^ dysregulation, and we found that δ-TT (15 μg/mL)-induced oxidative stress (12 h) in A375 and BLM cell lines was partially but significantly blocked by pretreatment with both 2APB (20 µM, 2 h) and DIDS (75 µM, 2 h) (Fig. 10a, b), supporting the existence of a close cause-effect relationship between the two processes. Indeed, these data highlight that the blockade of Ca^2+^ release from ER and the inhibition of its influx into mitochondria can prevent the mitochondrial ROS generation induced by δ-TT, suggesting that Ca^2+^ influx into mitochondria acts upstream of ROS overproduction in δ-TT-related paraptotic cell death.

## Discussion

Despite recent improvements in melanoma treatment, this tumor remains the leading cause of death among skin cancers [1]. Indeed, inherent or acquired drug resistance frequently leads to therapeutic failure, with patients experiencing tremendous adverse effects in the meanwhile [3–7]. Therefore, the identification of new anti-melanoma compounds and the development of novel treatment strategies are urgently needed.

Currently, many studies are focused on the paraptosis induced by natural products in cancer cells [11]. δ-TT, a vitamin E derivative endowed with powerful anti-tumor properties [18–22], has been shown to trigger this type of alternative cell death in colon and prostate cancer [24–26]. Herein, we investigated whether this compound could activate the paraptotic cascade in melanoma cells. We found that, in accordance with the main paraptotic features [10], δ-TT could cause extensive cytoplasmic vacuolization due to ER/mitochondrial enlargement in both A375 and BLM cell lines. Moreover, in line with the observations reported by Sperandio et al. [10, 12], vacuole formation could be successfully inhibited by pretreatment with the protein synthesis inhibitor cycloheximide, while the pan-caspase inhibitor Z-VAD-FMK only partially abolished δ-TT-related cytotoxicity; furthermore, pJNK, pP38 and pERK1/2 upregulation was observed, confirming the induction of paraptosis by δ-TT.

Given the wide disruption of mitochondrial structural integrity evidenced in treated cells, we examined δ-TT effects on the function of these organelles. Intriguingly, treatment of A375 and BLM cells with the nutraceutical resulted in OXPHOS impairment, with complex I downregulation, decreased oxygen consumption and loss of MMP; a significant reduction in ATP synthesis, culminating in AMPK activation, was also highlighted. Remarkably, various phytochemicals, including green tea, chrysin, silibinin, resveratrol and curcumin, have been reported to affect mitochondrial function in several tumor types, such as lung, breast and ovarian cancer [34–38]. However, literature data addressing the role of mitochondrial metabolism in the anti-tumor effects of TTs are still poor. Wang et al. have recently reported that γ-TT can inhibit OXPHOS in gastric adenocarcinoma cells by targeting mitochondrial complex I and II, while δ-TT suppressed mitochondrial activity and ATP production in HER-2 overexpressing breast cancer [39, 40]. We have also shown that mitochondrial structural and functional impairment is deeply implicated in δ-TT anti-prostate cancer activity [26]. In this context, our results not only support previous findings about the mitochondria-targeting ability of TTs in tumors but also suggest that δ-TT can severely alter the homeostasis of both ER [23] and mitochondria in melanoma cell lines.

Since mitochondrial dysfunction is often followed by oxidative stress [41], we explored the capability of δ-TT to impair the redox balance of A375 and BLM human melanoma cells. Indeed, it was demonstrated that this compound can boost cytotoxic ROS production in breast and prostate cancer models [26, 40]. In melanoma cell lines, we discovered that it could trigger mitochondrial ROS generation, and that NAC-mediated ROS scavenging could counteract its effects on cell viability, vacuole formation and MAPK activation, highlighting the involvement of oxidative stress in the paraptosis evoked by δ-TT. These results are consistent with previous reports describing the crucial role of ROS signaling in the paraptotic cell death triggered by different natural products, such as ginsenosides, honokiol, chalcomoracin and withaferin A, in several malignancies [42–46].

Based on the above evidence, we finally dissected the potential pathways responsible for the paraptotic cascade observed during δ-TT treatment in melanoma cells. It is well-known that the ER and mitochondria are the main regulators of Ca^2+^ levels in the cell and that the transfer of this ion between them occurs through the mitochondria-associated ER membranes (MAMs) [47]. VDAC and IP3R are key regulators of Ca^2+^ permeability in MAMs [48]. In our study, δ-TT triggered both cytoplasmic and mitochondrial Ca^2+^ overload, and pretreatment with 2APB, an IP3R inhibitor, and DIDS, a VDAC blocker, effectively suppressed δ-TT-related cytotoxicity. In addition, the inhibition of Ca^2+^ release from ER and of its influx into mitochondria markedly reduced ER/mitochondrial swelling and MAPK phosphorylation, highlighting its role in the paraptotic alterations evidenced in treated cells. Finally, both 2APB and DIDS prevented mitochondrial ROS overproduction, thus evidencing the contribution of the Ca^2+^/ROS axis to δ-TT-stimulated paraptotic cell death in A375 and BLM cell lines. Notably, several natural compounds have been shown to trigger Ca^2+^ dysregulation-related paraptotic death in tumors [31, 32, 49]; among them, morusin and hesperidin have been found to specifically mediate a cytotoxic interplay between Ca^2+^ and ROS signaling [28, 29]. To our knowledge, this is the first study reconstructing the crucial role of these molecular interactions in the anti-melanoma effects of TTs.

In conclusion, our findings highlight the importance of mitochondria as targets for the development of new anti-cancer strategies and offer a deeper understanding of the anti-melanoma activity of δ-TT, pointing out that it can induce Ca^2+^/ROS-associated mitochondrial dysfunction-dependent paraptosis in A375 and BLM cell lines.

## Data Availability

Data supporting the findings of this study are available from the corresponding author, PL, upon reasonable request.
